# Carbon Transformation Induced by High Energy Excimer Treatment

**DOI:** 10.3390/ma15134614

**Published:** 2022-06-30

**Authors:** Nikola Slepičková Kasálková, Klaudia Hurtuková, Dominik Fajstavr, Ladislav Lapčák, Petr Sajdl, Zdeňka Kolská, Václav Švorčík, Petr Slepička

**Affiliations:** 1 Department of Solid State Engineering, University of Chemistry and Technology, 166 28 Prague, Czech Republic; nikola.kasalkova@vscht.cz (N.S.K.); klaudia.hurtukova@vscht.cz (K.H.); dominik.fajstavr@vscht.cz (D.F.); vaclav.svorcik@vscht.cz (V.Š.); 2 Central Laboratories, University of Chemistry and Technology, 166 28 Prague, Czech Republic; ladislav.lapcak@vscht.cz; 3 Department of Power Engineering, University of Chemistry and Technology, 166 28 Prague, Czech Republic; petr.sajdl@vscht.cz; 4 Faculty of Science, J. E. Purkyně University in Ústí nad Labem, 400 96 Ústí nad Labem, Czech Republic; zdenka.kolska@ujep.cz

**Keywords:** excimer laser, carbon, surface morphology, surface chemistry, Raman analysis, amorphous carbon, Q-carbon

## Abstract

The main aim of this study was to describe the treatment of carbon sheet with a high-energy excimer laser. The excimer modification changed the surface chemistry and morphology of carbon. The appearance of specific carbon forms and modifications have been detected due to exposure to laser beam fluencies up to 8 J cm^−2^. High fluence optics was used for dramatic changes in the carbon layer with the possibility of Q-carbon formation; a specific amorphous carbon phase was detected with Raman spectroscopy. The changes in morphology were determined with atomic force microscopy and confirmed with scanning electron microscopy, where the partial formation of the Q-carbon phase was detected. Energy dispersive spectroscopy (EDS) was applied for a detailed study of surface chemistry. The particular shift of functional groups induced on laser-treated areas was determined by X-ray photoelectron spectroscopy. For the first time, high-dose laser exposure successfully induced a specific amorphous carbon phase.

## 1. Introduction

At present, a large part of industry and research dealing with material properties is focused on the properties of carbon, which determine the behavior of all carbon materials. For this reason, 2004 was an important year in material science, which led to the discovery of a new carbon structure—graphene [1]. It is a 2D monolayer of carbon atoms with sp2 hybridization arranged in a hexagonal lattice. This different arrangement of graphene gives its material unique properties, and therefore it has become the target of numerous studies with increased interest. This research aims to study the already excellent properties of graphene in the physical, biological, and mechanical fields, such as flexibility, mechanical strength, transparency, thermal conductivity, electrical conductivity, tunable optical properties, and intrinsic mobility [2,3,4,5,6,7]. These properties can then be enhanced and modified in the preparation of graphene in combination with different nanostructures. This is also proven by numerous studies presenting the possibility of using graphene for the production of transistors, sensors, or supercapacitors [8,9,10]. This work, which focuses on the preparation of supercapacitor electrodes made with graphene, shows a much better performance of these structures in disordered form compared to activated carbon or graphene in an ordered form due to the larger surface area. It is ensured by randomly arranged pores and by greater access of ions to the volume [11].

Historically, the first preparation of graphene was carried out by the method of mechanical exfoliation, but the organic residues from the tape required subsequent thermal stress to clean the surface and achieve an uncontaminated sample [1]. In contrast, chemical exfoliation is based on the oxidation of graphite and its reduction to graphene, which, however, creates defects on the surface of the material [12]. For this reason, several new methods for preparing graphene have been proposed.

One way to synthesize graphene is to use laser radiation to ablate carbon and achieve carbon monolayers. The advantage of the laser process is that it is not necessary to work at high temperatures, pressures, or using acids and bases that negatively affect the environment. On the contrary, it is a fast, direct, and clean procedure for modifying a sample using monochromatic coherent radiation. The preferred approach seems to be the choice of an excimer laser operating in a pulsed mode and with a short pulse duration (ns), which reaches a power in the order of megawatts. The short pulse duration is crucial because the heat released during the pulse does not have enough time to penetrate from the surface into the lower layer. This laser modification of short and high-power pulses is often used today for precise surface treatment without damaging the bottom layer, which is also used in medicine for eye surgery or in the restoration of artworks [13].

Recent studies of laser ablation of carbon materials in the form of graphene are currently underway and present different ambient conditions such as gaseous, liquid, or vacuum environments [14,15,16]. Graphene exfoliation achieved by pulsed laser ablation in a liquid medium is described in [17], where graphite, flexible graphite, and pyrolytic graphite are used as targets. Important parameters of the process are the wavelength of the laser, the type of liquid medium, and the target to achieve high-quality graphene nanolayers. Synthesis using a nanosecond laser pulse irradiating flexible graphite, which rapidly leads to high-quality layers during one exfoliation step, proved to be the most optimal choice of parameters for the synthesis of graphene in a liquid medium [18].

The use of pulsed laser exfoliation in a vacuum environment to obtain 2D carbon materials from a polymer matrix was described in 2018 [19]. A polymer was deposited as a substrate in a vacuum chamber, and the surface was irradiated with a 532 nm Nd: YAG pulsed laser to peel the surface layers. The exfoliated material in the form of 2D carbon layers (CNS) then covered the substrate. Other studies are exploring ways to use other materials for laser exfoliation. In general, suitable conditions are proposed for laser exfoliation, such as a flat surface of the irradiated material, a suitable depth of ablation, a non-oxidizing environment, and also lower energy of the incident photon from the laser beam than the binding energy in the C-C structure. Theoretical works deal with simulations of possible processes during laser modification and the influence of wavelength and energy of the used laser beam [20]. For example, a study by Smirnov et al. is known for setting a wavelength condition. It determines that for GO reduction by a photochemical effect, the incident photon must reach a wavelength of less than 387 nm [21]. For this reason, an excimer beam with a lower wavelength and a higher pulse power in the order of megawatts cleaves all the bonds connected to the GO [22]. This cleavage of functional groups and the breaking of bonds is the result of a process of nonlinear photon absorption, where two or more photons are absorbed into a chemical bond, which is subsequently disrupted. The consequence of this destruction of bonds can be an increase in porosity but also the destruction of optical instruments [23].

Laser modification can also be used in the production of thin-film micro capacitors, where an excimer laser is used to reduce graphite oxide. The advantage of this laser reduction is its timesaving compared to the reduction of graphene oxide by microwaves [24]. This method of easy and rapid synthesis of graphene provides more homogeneous nanolayers, without surface oxygen, and is more continuous than in the case of microwave reduction or other rapid methods. Under the given conditions, laser irradiation of graphene leads, in addition to reduction and exfoliation, also to an increase in the porosity of the layer, which can be used in the preparation of micro capacitor elements [25].

At present, however, attention is focused on a new phase of carbon, named quenched carbon (Q-carbon, Q-C), which shows excellent ferroelectric-related properties, even superior to diamond [26]. Q-carbon, discovered in 2015, is a recently discovered metastable phase of carbon [27]. It is formed by converting amorphous carbon thin films by nanosecond pulsed laser melting and subsequent quenching in a super undercooled state [28]. It has been proposed that liquid carbon may exist as a thermodynamically stable phase at high pressures and temperatures. Depending on the laser energy density and the physical properties of the amorphous film and the substrate, a process of super undercooling and quenching takes place, which leads to the formation of a metastable state of amorphous Q-carbon structure possessing unique properties [29]. It is shown that nanosecond laser heating of diamond-like amorphous carbon on sapphire, glass, and polymer substrates can be confined to melt carbon in a super undercooled state [29].

Although there are some experimental studies of the effect of laser fluence values and laser wavelengths on carbon structures and their formation, the interaction of a high fluence excimer laser with carbon paper consisting of thin amorphous nanoflakes has not been studied so far. The aim of this work is to study the effect of laser modification with respect to physico-chemical changes, structural changes, and the appearance of new carbon forms. The transformations of carbon sheet in a high vacuum, the possibility of changes in carbon hybridization, and possible new carbon forms amid its high-energy transformation connected with melting or quenching are presented. To our best knowledge, this topic has not been studied yet and is thus the target of our research.

## 2. Materials and Methods

### 2.1. Materials and Laser Treatment

We used commercially available carbon foil (75 μm thick foil, crystal structure Hexagonal/Diamond, melting point 3650 °C supplied by Goodfellow Ltd., Huntingdon, UK). Surface of carbon foil was modified with a high energy pulsed excimer KrF laser (Coherent Inc., Santa Clara, CA, USA, Leap 100 K) with a wavelength of 248 nm and a pulse duration of 20–40 ns, repetition rate of 1 Hz) with stabilized energy range 900–1000 mJ and beam dimensions of 32 × 13 mm^2^. An aperture of 30 × 10 mm^2^ used in the experiments with the application of a lens with the ability to increase the laser fluence up to 8000 mJ·cm^−2^.

### 2.2. Characterization

The surface morphology and roughness of modified polymer samples were examined by atomic force microscopy (AFM) and laser confocal microscopy. For AFM analysis, we used Dimension ICON (Bruker Corp., Billerica, MA, USA), ScanAsyst mode in air was used for determination. Silicon tip on nitride lever SCANASYST-AIR with spring constant 0.4 N m^−1^ was used. NanoScope Analysis software (version 1.80, Bruker Corp., Billerica, MA, USA) was applied for data processing. The mean roughness value (Ra) represents the average of the deviations from the center plane of the sample. For the laser confocal microscopy (LCM) analysis, the Olympus LEXT OLS 3100 microscope (Olympus Czech Group, s.r.o., Prague, Czech Republic) was chosen with the blue laser (λ = 407 nm) and with a magnification of up to 14,400×.

The surface morphology and elemental composition were acquired with a scanning electron microscope (SEM) and energy-dispersive X-ray spectroscopy (EDS). We used the LYRA3 device (Tescan, Brno, Czech Republic) with an applied acceleration voltage of 10 kV for SEM and F-MaxN analyzer and SDD detector (Oxford Instruments, Abingdon, UK) with an applied acceleration voltage of 10 kV for EDS.

The presence and oxidation state of carbon and partial presence of oxygen in the surface layer was determined by X-ray photoelectron spectroscopy (XPS). An Omicron Nanotechnology ESCAProbeP spectrometer (Omicron nanotechnology GmbH, Taunusstein, Germany) was used. The exposed and analyzed area had a dimension of 2 × 3 mm^2^. The X-ray source was monochromatic at 1486.7 eV. Atomic concentrations of elements were determined by the CASA XPS program using an integrated area of spectrum lines and relative sensitivity factors, which are quoted in the database of CASA XPS. The samples were analyzed under a take-off angle of 19°.

Raman analysis was performed on a dispersed Raman spectrometer DXR Microscope of the company Thermo Scientific (Walthamm, MA, USA) equipped with confocal microscope Olympus and thermoelectrically cooled CCD detector. As an excitation source, we used solid-state Nd:YAG laser (wavelength 532 nm, maximum power 10 mW). Grating with 900 lines/mm, 25 µm slit aperture, and 50× magnification objective was utilized. Measurement conditions for the samples were 10 mW laser power, 10 s acquisition time per scan, and 20 repetitions. Ten spectra were averaged from each surface. Data were processed using Omnic 9 software (Thermo Scientific, Walthamm, MA, USA).

## 3. Results and Discussion

### 3.1. Surface Morphology and Roughness

As was referred above, in this study, we have focused on the possibility of high-temperature transformations of carbon foil by high-energy excimer exposure. This transformation was studied mainly from the point of view of surface chemistry and surface morphology changes. As was published earlier, for lower carbon thicknesses of several hundreds of nanometers, the high-temperature transformation to Q-carbon was successfully prepared, which showed excellent ferroelectric-related properties, even superior to diamond [26]. Q-carbon, discovered in 2015, is a recently discovered metastable phase of carbon [27]. It is formed by converting amorphous carbon thin films by nanosecond pulsed laser melting and subsequent quenching in a super undercooled state [28,29,30]. Further, recent knowledge about transformations regarding the sp2/sp3 original ratio and its impact on the Q-carbon was published in [31].

**Figure 1:**
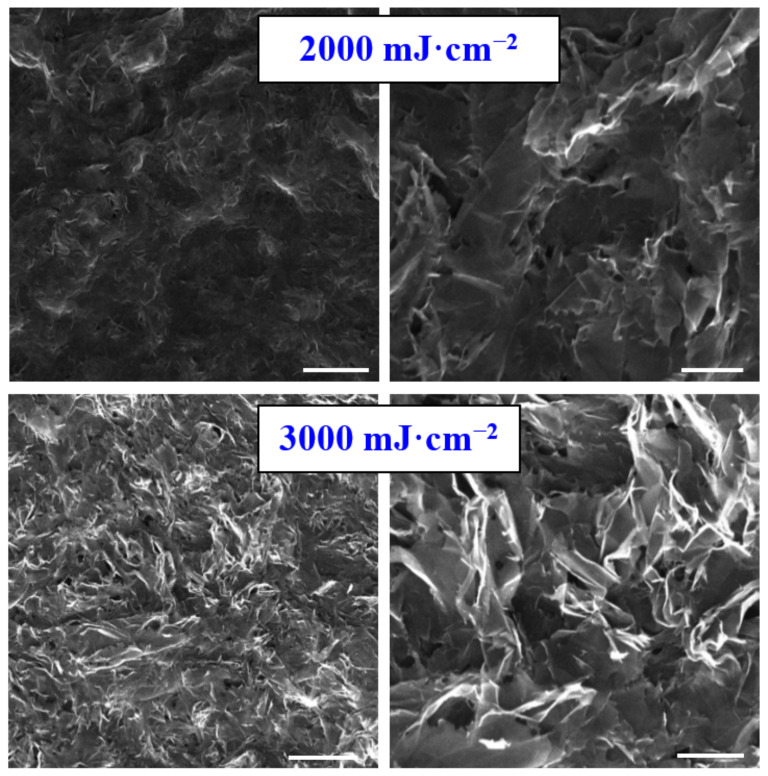
Scanning electron microscopy images of carbon sheet exposed with high-energy excimer laser and laser fluencies for 2000 and 3000 mJ·cm^−2^. The scanning areas were 10 × 10 µm^2^ (**right** column) and 30 × 30 µm^2^ (**left** column). White lines represent six microns (**left**) and two microns (**right**).

**Figure 2:**
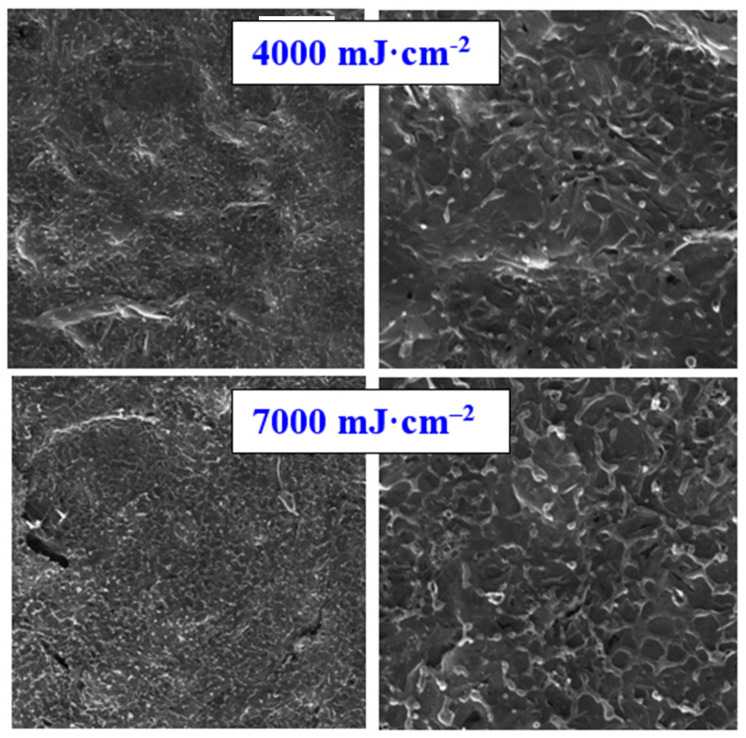
Scanning electron microscopy images of carbon sheet exposed with high-energy excimer laser and laser fluencies for 4000 and 7000 mJ·cm^−2^. The scanning areas were 10 × 10 µm^2^ (**right** column) and 30 × 30 µm^2^ (**left** column). White lines represent six microns (**left**) and two microns (**right**).

The first step in the study of high-energy excimer impact on carbon sheet was to characterize the surface morphology changes. Pristine carbon foil consists of nanoflakes with a thickness of several tenths of nanometers, the morphology of which is similar to that presented in Figure 1 for excimer treatment with 2000 mJ·cm^−2^; therefore, we did not introduce the surface morphology of pristine one. As it is obvious from Figure 1 and Figure 2, carbon transformation is induced only for high laser fluencies, above 4000 mJ·cm^−2^. The laser fluence 3000 mJ·cm^−2^ induced partial “opening” of the nanosheets, but local melting is still not present. The high-energy induced carbon melt was firstly achieved for the fluence 4000 mJ·cm^−2^ and persisted for all higher fluencies. Due to the application of the excimer laser wavelength, we can observe a very interesting phenomenon—that the surface melting occurs on the very surface of the carbon foil; thus, the bulk of the foil remains almost unaffected. The melting-induced network structure with specific spherical ends are more visible on the left column (detailed images of the samples).

**Figure 3:**
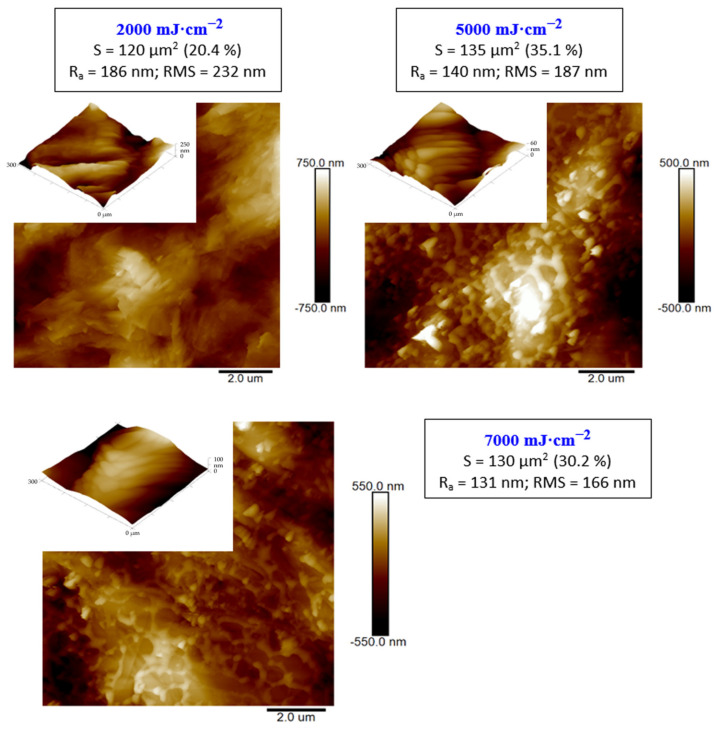
Atomic force microscopy images of carbon sheet exposed with high energy excimer laser and laser fluencies from 2000 to 7000 mJ·cm^−2^. The scanning areas were 10 × 10 µm^2^. R_a_ represents the average surface roughness, RMS root mean square roughness, integral area (S), and the difference from the basic area. The inset represents the 300 × 300 nm^2^ square detail of the morphology.

As the alternative method for surface morphology changes, atomic force microscopy was applied. If we look closely at the surface nanostructure of the carbon foil, we have determined a similar combination of carbon nano leaf after the melting process, as was determined with the SEM technique. As it is obvious from Figure 3, the laser treatment 2000 mJ·cm^−2^ did not change the surface morphology significantly, which is in good agreement with scanning electron microscopy results. The increased laser fluence induced the carbon leaf formation “collapse”, melting, and formation of the network of super cooled carbon, as it is described on the 2nd and 3rd lines of Figure 3. As it is obvious from this Figure, with increasing laser fluence above the carbon melt threshold, there are no significant further changes in the carbon morphology, including surface properties, such as roughness or effective surface area; however, these results can be applied only for application of single laser shot, and the roughness/morphology is significantly altered if more laser pulses are applied. As it is obvious from the insets of three images presented in Figure 3, the melted highly amorphous carbon consists of specific layers combined layer by layer with a thickness of several tenths of nanometers.

Excimer exposure with nanosecond pulses has been successfully applied for the treatment of graphite oxide [32] or femtosecond laser pulses were used for simultaneous nanopatterning and reduction of graphene oxide [33], where self-organized periodic structures of reduced graphene oxide (rGO) appeared on GO surfaces upon processing with a femtosecond laser at fluencies slightly higher than the fluence needed for reduction of the GO. The interaction of carbon with plasma or excimer laser was previously described in [34,35,36,37,38,39,40,41]. The interesting chemical and morphological changes in the carbon sheets have been induced. The second step was to determine the changes in surface chemistry. For this reason, two analytical methods were applied, EDS and XPS.

### 3.2. EDS Analysis

**Figure 4:**
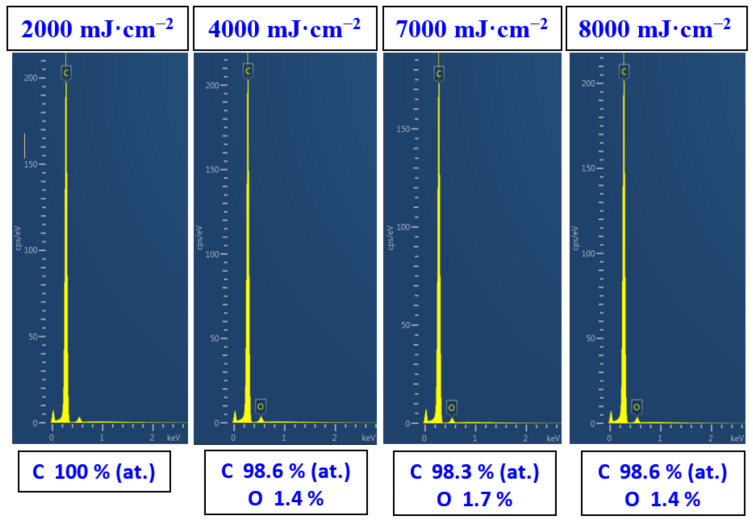
Energy dispersive spectra of carbon sheets exposed with high energy excimer laser and laser fluencies from 2000 to 8000 mJ·cm^−2^.

The changes in surface chemistry due to excimer treatment determined by EDS revealed only minor changes in oxygen concentration of carbon foil. The pristine carbon did not exhibit any oxygen on its surface; the same result was observed for exposure with 2000 mJ·cm^−2^. A minor increase in oxygen concentration was observed for laser fluencies above 3000 mJ·cm^−2^; however, no significant trend for particular laser fluencies was observed. The slight oxidation was probably induced after the removal of the samples from the vacuum chamber into the ambient atmosphere. The atomic concentration of oxygen did not exceed 2 at.% according to the EDS analysis (Figure 4); therefore, we can conclude that due to the transformation of carbon under higher fluencies, the melted layer remains stable with no significant chemical changes. This is due to the fact that the treatment is conducted in a relatively high vacuum, better than 10^−5^ Pa, which enables the preservation of the character of the carbon in a chemically unchanged form, while remaining structurally altered.

**Figure 5:**
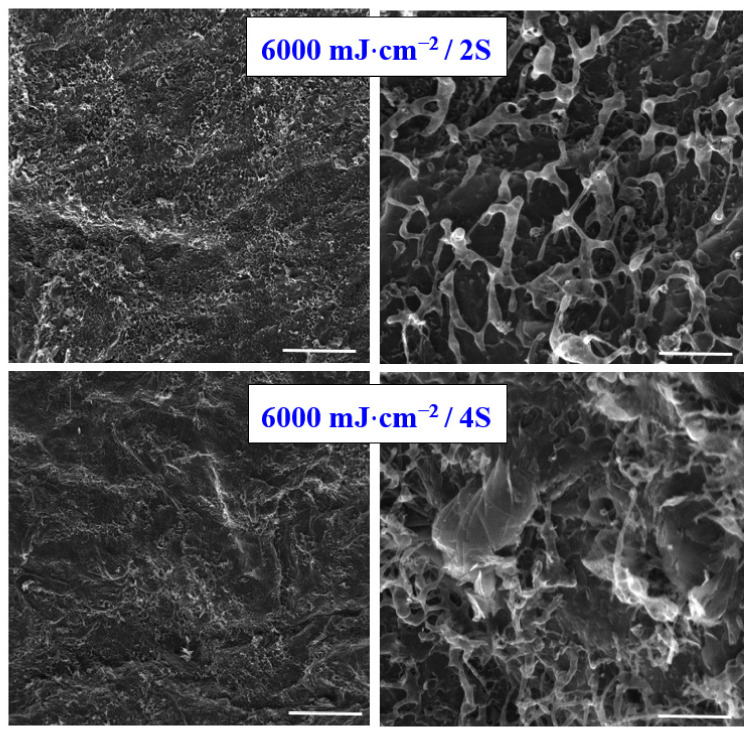
Scanning electron microscopy images of carbon sheet exposed with high energy excimer laser and laser fluence 6000 mJ·cm^−2^, two (2S) and four shots (4S) were applied. The scanning areas were 100 × 100 µm^2^ (**left** column) and 10 × 10 µm^2^ (**right** column). White lines represent 20 microns (**left**) and 2 microns (**right**).

On the basis of results acquired from the single-shot excimer laser study, we continued the study for a higher number of laser pulses. We selected the laser beam fluence 6000 mJ·cm^−2^, and we selected the number of laser pulses two and four. Our main aim was only to estimate if some significant additive changes regarding carbon structure and properties occurred if an even higher laser dose was applied. As it is obvious from Figure 5, the melting process connected with the formation of an amorphous carbon network on the treated surface is also preserved for higher laser doses; however, as it is obvious from Figure 5, the surface physico-chemical changes followed the trend from previous treatment with a single shot, no “significant side effect” has been observed. The roughness of the pattern is increased; however, no additional nano or microstructures have been observed; therefore, we concluded not to conduct further experiments by increasing the number of laser beam shots and only to further analyze the pattern constructed by single-shot exposure.

### 3.3. Raman Spectroscopy

**Figure 6:**
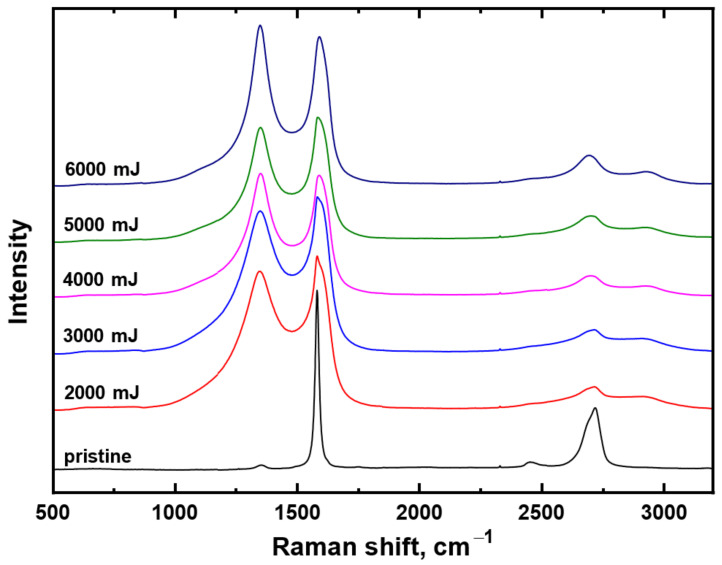
Average Raman spectra for each characterized sample—pristine carbon sheet and the substrate exposed with single-shot excimer with laser fluence ranging from 2000 mJ·cm^−2^ to 6000 mJ·cm^−2^.

The important structural properties of carbon can be revealed with Raman spectroscopy; therefore, we introduce the Raman spectrum of pristine carbon (Figure 6, black line) and particular carbon spectra for samples treated with a single shot and different laser fluence in the interval from 2000 mJ·cm^−2^ to 6000 mJ·cm^−2^ (Figure 6). From these spectra, it is evident that, due to high energy exposure, the characteristic peak at the position 1350 cm^−1^ appeared. In the spectra, a typical structure for disordered carbon structure can be found. It is a combination of two bands known as the D band (maximum at ~1310 cm^−1^) reflecting the presence of defects in samples and the G band (maximum at ~1530 cm^−1^). Generally, Raman spectra of amorphous, nanostructured, diamond-like carbon, and nanodiamond were discussed in [42] and above spoken bands were reported for graphite [43] and by Couzi et al. [44] for the heat-treated glassy carbons and graphite nanoplatelets. Averaged Raman spectra of differently treated samples show a similar ratio I_D_/I_G_ ~ 1, similarly to the spectra of above-mentioned carbon allotropes. A higher I_D_/I_G_ ratio corresponds to more disordered domains.

The Raman spectrum of pristine carbon paper corresponds to the Raman spectrum of graphite with a relatively small number of defects, i.e., quasi-perfectly ordered three-dimensional (3D) carbon [44,45]. Spectral interpretation of the main Raman bands was carried out in accordance with generally accepted assignment in the literature for ordered and disordered graphitic carbons [46,47]. The most prominent feature in the Raman spectrum of graphite is the G band at 1581 cm^−1^, which corresponds to the E_2g_ phonon mode at the center of the Brillouin zone and is usually present in all graphitic materials [44]. The shift in a position towards higher wavenumbers with increasing modification power was published in [48]. The pristine spectrum of nanographite paper also exhibits the second-order (D + D”), 2D, and 2D’ Raman peaks situated at wavenumber 2452 cm^−1^, 2718 cm^−1^, and 3246 cm^−1^, respectively (the 2D’ band is not shown in Figure 5). It is generally accepted that the 2D and 2D’ bands are the higher harmonics of Raman inactive vibrational modes in ordered carbon materials, which are enhanced through double resonance processes involving the creation of two phonons with opposite wave vectors [44].

We can therefore summarize that our pristine material called nanographite carbon paper provided a similar Raman spectrum to highly organized graphite; only the 2D peak was slightly shifted toward position 2700 cm^−1^ compared to the spectrum of highly oriented pyrolytic graphite in [44].

A rather different situation occurred after exposure to a high-energy excimer laser; the spectra are introduced within Figure 6 for different laser beam fluencies. The significant morphology changes as was discussed above, but also structural changes occurred in the laser-treated carbon and new features appeared on the Raman spectrum, as it is evident from Figure 6. In particular, the laser treatment has led to the presence of the fundamental D band situated around 1345 cm^−1^ and to the broadening of the G band. The appearance of a new fundamental peak is caused by the partial disorder induced by high-energy laser annealing that creates holes in the π valence band. The electron–hole mechanism then enables the single phonon double resonance scattering outside the center of the Brillouin zone. The D band position in the spectra of laser-treated samples is slightly shifted from that reported in [44]. This shift corresponds to the dispersive nature of the D band and the different excitation lasers used. Another phenomenon is that the excimer laser induces the diminishing of the second-order (D + D”) and 2D’’ peaks, while the signal at position approx. 2700 cm^−1^ is lowered (peak 2D). A new broad second-order Raman peak is present at position approx. 2930 cm^−1^. It is activated by the defects through the electron–hole interaction, similarly to the D band and it is assigned to the combination D + G or D + D’ [44].

**Figure 7:**
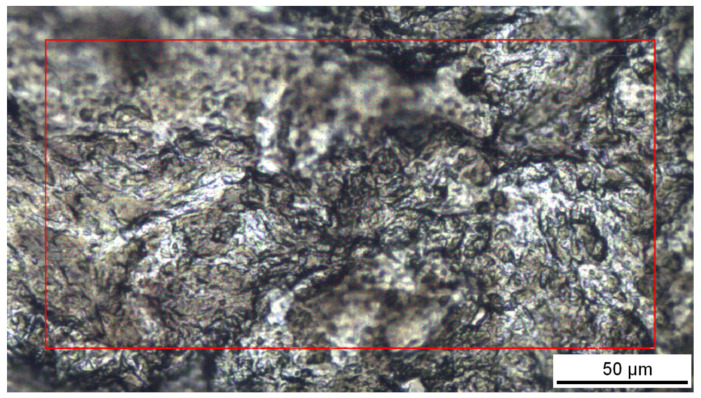
Optical image of carbon sample treated with excimer laser 4000 mJ·cm^−2^.

**Figure 8:**
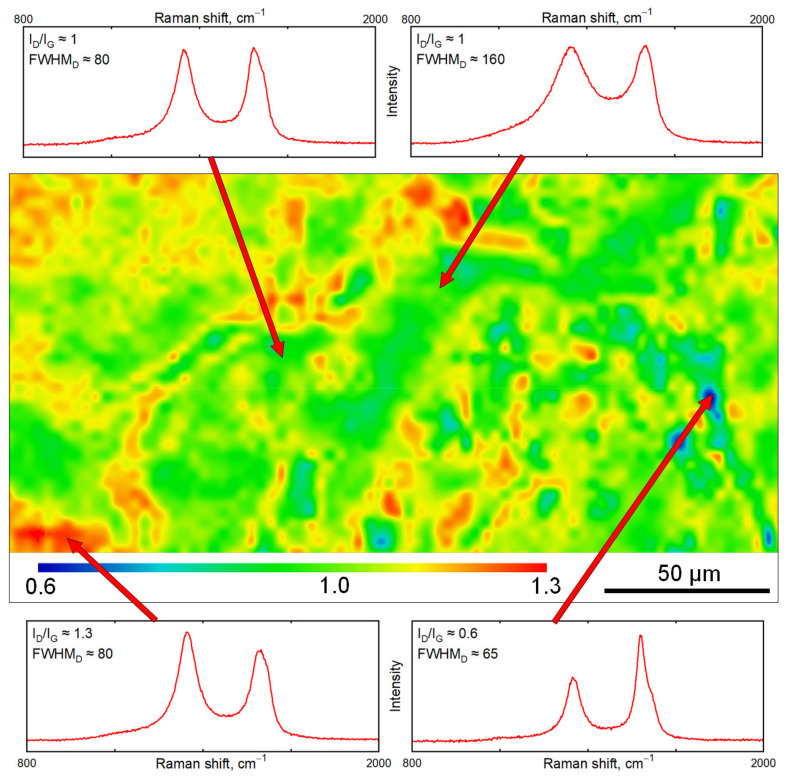
I_D_/I_G_ contour map of carbon sample treated with excimer laser 4000 mJ·cm^−2^ with selected type spectra.

The 10 measured points on irradiated samples exhibited quite a high variance of Raman spectra; therefore, in order to examine the surface homogeneity, Raman mapping of carbon sheet exposed with a laser fluence of 4000 mJ·cm^−2^ was performed. The image from the optical microscope of the laser-treated sample is introduced in Figure 7, while the Raman contour plot together with selected typical Raman spectra is shown in Figure 8. The contour plot colors are based on the ratio of the intensities of the D band and G band (I_D_/I_G_). Probably due to the unique structure of pristine carbon nanosheet (crystal structure hexagonal/diamond), some different areas can be distinguished on the treated surface on the basis of Raman mapping. The major part of the analyzed laser-treated area can be described as disordered carbon with I_D_/I_G_ ratio close to 1, but also with different FWHM; however, considerable areas with I_D_/I_G_ up to 1.3 or I_D_/I_G_ down to 0.6 have also been induced by the excimer. In general, the area with a higher I_D_/I_G_ ratio corresponds to more disordered domains; by contrast, the lower I_D_/I_G_ ratio indicated more organized domains with some spectra resembling the graphitic phase. The dependency of the I_D_/I_G_ ratio versus the degree of disorder is not monotonic, so there is the possibility of differently disordered materials with the same I_D_/I_G_ value; therefore, it is also necessary to take the width of Raman bands into account. In the Raman mapped surface can be distinguished spectra with similar I_D_/I_G_ ratio but with different peak widths between 80–160 cm^−1^, which suggests further inhomogeneity of the irradiated surface. Summary of possible carbon forms and corresponding Raman spectra with respect to the particular peak positions were discussed in detail in [42].

From the Raman analysis, we can finally conclude that the high laser fluence applied to the carbon layer leads to the recrystallization of the carbon surface; melting and rapid cooling lead to the formation of disordered carbon with similar Raman characteristics and peak positions similar to the heat-treated glassy carbon or graphite nanoplatelets described in [44]. However, the peak positions and rapid carbon melting could also suggest a presence of DLC layers with similar Raman characteristics and peak positions similar to amorphous DLC described in [49] or Q-carbon [26,27,28,29,30,31], so Q-carbon formation at the edges of the domains is probable, the G peak position (up to 1575 cm^−1^) was higher than the peak position for the vibrational mode of graphic planes (1540 cm^−1^). Properties of the different carbon materials were described in detail in [50]. The above spoken Raman characteristics indicated the presence of compressive strain in the film [51], supporting the formation of Q-carbon [31]. The chemical and surface changes induced by excimer laser wavelength were also observed for silicon and semiconductors [52,53] or silicon carbide [54,55,56]. The discussion on the correlation of surface morphology (e.g., SEM and AFM images) and I_D_/I_G_ ratio changes were included in the papers [57,58].

### 3.4. X-ray Photoinduced Spectroscopy

**Figure 9:**
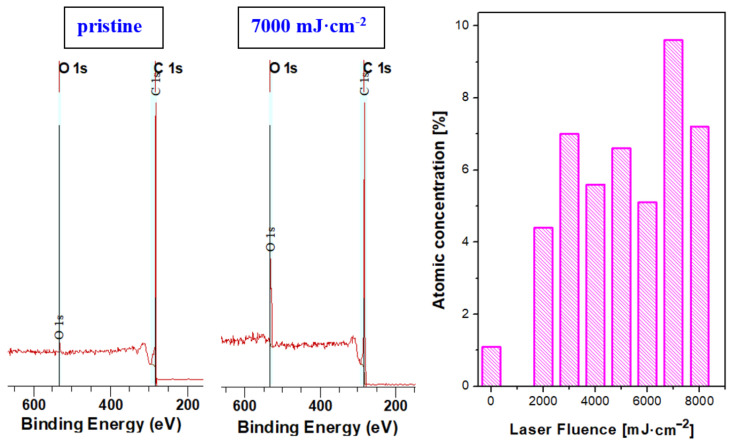
Selected XPS spectra of pristine carbon foil and carbon foil exposed with an excimer laser and fluence 7000 mJ·cm^−2^. On the left, the oxygen surface atomic concentrations for pristine carbon foil and carbon foil treated in interval from 2000 mJ·cm^−2^ to 8000 mJ·cm^−2^.

The surface chemistry of up to 10 atomic layers of the treated surface was studied with X-ray photoelectron spectroscopy; the selected spectra of pristine carbon and laser-treated carbon are introduced in Figure 9. It is obvious that even pristine carbon nanopaper possesses a minor amount of oxygen on its surface. The high-energy laser treatment increases the oxygen concentration, as it is described in the right part of Figure 8. As it is obvious from this dependence, the excimer laser treatment increases the oxygen concentration; however, only oxygen incorporation after removal from the vacuum chamber is expected.

## 4. Conclusions

We have exposed the carbon paper with hexagonal/diamond crystal structure by excimer exposure, which changed its surface chemistry and morphology significantly. The appearance of specific carbon forms and modifications have been detected due to exposure to laser beam fluencies up to 8000 mJ·cm^−2^. Carbon transformation was induced only for high laser fluencies above 4000 mJ·cm^−2^. Due to the application of excimer laser wavelength, we can observe a very interesting phenomenon—that the surface melting occurs on the very surface of the carbon foil; thus, the bulk of the foil remains almost unaffected. The melting induced a network structure with specific spherical ends. The oxygen concentration in the top 10 atomic layers was altered with an increase in oxygen up to 9.4%, which was detected with XPS analysis; however, EDS did not confirm significant changes in oxygen concentration for the modified surface (up to several hundreds of nm), which supports only partial oxidation of the surface after removal from the chamber. The structural changes induced by laser treatment were confirmed by Raman analysis; the laser exposure led to the broadening of the G band, while new peaks appeared on the Raman spectrum. Laser treatment has led to the presence of the D band situated at 1345 cm^−1^, which is induced by the partial disorder induced with high-energy laser annealing. The excimer laser also induces diminishing of the (D + D”) and 2D’ peaks, while the 2D band at position approx. 2700 cm^−1^ is lowered and a new broad second-order Raman peak appears at position approx. 2930 cm^−1^ activated as the result of defects formed in the crystal structure. The peak positions and rapid carbon melting could also suggest a presence of DLC layers with similar Raman characteristics and peak positions similar to amorphous DLC or Q-carbon, so the Q-carbon formation at the edges of the domains is probable; the G peak position (up to 1575 cm^−1^) was higher than the peak position for the vibrational mode of graphic planes (1540 cm^−1^). This indicated the presence of compressive strain in the film, supporting the formation of Q-carbon.

## Data Availability

Data are contained within the article.
